# Plasma Docosahexaenoic Acid and Eicosapentaenoic Acid Concentrations Are Positively Associated with Brown Adipose Tissue Activity in Humans

**DOI:** 10.3390/metabo10100388

**Published:** 2020-09-28

**Authors:** Angie S. Xiang, Corey Giles, Rebecca K.C. Loh, Melissa F. Formosa, Nina Eikelis, Gavin W. Lambert, Peter J. Meikle, Bronwyn A. Kingwell, Andrew L. Carey

**Affiliations:** 1 Metabolic and Vascular Physiology Laboratory, Baker Heart and Diabetes Institute, Melbourne 3004, Australia; Angie.Xiang@monash.edu.au (A.S.X.); Rebecca.Loh@baker.edu.au (R.K.C.L.); Melissa.Formosa@baker.edu.au (M.F.F.); Bronwyn.Kingwell@baker.edu.au (B.A.K.); Andrew.Carey@baker.edu.au (A.L.C.); 2 Central Clinical School, Monash University, Clayton, Melbourne 3004, Australia; 3 Metabolomics Laboratory, Baker Heart and Diabetes Institute, Melbourne 3004, Australia; Peter.Meikle@baker.edu.au; 4 Department of Physiology, Monash University, Clayton, Melbourne 3800, Australia; 5 Iverson Health Innovation Research Institute, Swinburne Institute of Technology, Melbourne 3122, Australia; NEikelis@swin.edu.au (N.E.); GLambert@swin.edu.au (G.W.L.); 6 Research Therapeutic Area, CSL Limited, Parkville 3052, Australia

**Keywords:** fatty acids, fish oil, lipidomics, mass spectrometry, obesity, BAT, brown adipose tissue, oxylipin, NEFA, DHA, EPA

## Abstract

Brown adipose tissue (BAT) activation is a possible therapeutic strategy to increase energy expenditure and improve metabolic homeostasis in obesity. Recent studies have revealed novel interactions between BAT and circulating lipid species—in particular, the non-esterified fatty acid (NEFA) and oxylipin lipid classes. This study aimed to identify individual lipid species that may be associated with cold-stimulated BAT activity in humans. A panel of 44 NEFA and 41 oxylipin species were measured using mass-spectrometry-based lipidomics in the plasma of fourteen healthy male participants before and after 90 min of mild cold exposure. Lipid measures were correlated with BAT activity measured via ^18^F-fluorodeoxyglucose ([^18^F]FDG) positron emission tomography/computed tomography (PET/CT), along with norepinephrine (NE) concentration (a surrogate marker of sympathetic activity). The study identified a significant increase in total NEFA concentration following cold exposure that was positively associated with NE concentration change. Individually, 33 NEFA and 11 oxylipin species increased significantly in response to cold exposure. The concentration of the omega-3 NEFA, docosahexaenoic acid (DHA) and eicosapentaenoic acid (EPA) at baseline was significantly associated with BAT activity, and the cold-induced change in 18 NEFA species was significantly associated with BAT activity. No significant associations were identified between BAT activity and oxylipins.

## 1. Introduction

Brown adipose tissue (BAT) has been of interest for several decades as a potential anti-obesity target in humans—particularly after the recent conclusive identification of functional BAT and clarification of its location in adult humans [1,2,3,4,5,6]. This is due to BAT’s capacity for non-shivering thermogenesis, which contributes to increased whole-body energy expenditure. Recent studies have suggested that adult humans possess >1 kg of adipose tissue capable of thermogenesis (“brownable” adipose tissue) [1], the maximal activation of which could increase whole body energy expenditure by ~25% [7]. However, to date it has not been possible to increase BAT energy expenditure by this magnitude in humans through chronic administration of pharmacological agents [7,8,9,10]. The search for factors that could increase BAT browning and activity is therefore important and ongoing. Additionally, current techniques available for assessment of BAT activity in humans are costly and suffer technical challenges. Therefore, in addition to identification of endogenous activators, the discovery of novel biomarkers of BAT activity in humans is important for progress in this field.

Physiologically, BAT thermogenesis results from β-adrenergic stimulation of brown adipocyte lipolysis, initiating long-chain non-esterified fatty acid (NEFA) mobilization and accumulation within adipocytes. These long-chain NEFA outcompete inhibitory adenine nucleotides on the BAT thermogenic protein uncoupling protein-1 (UCP-1), leading to dissipation of the mitochondrial intermembrane electrochemical gradient, thereby increasing catabolism of substrates that normally sustain ATP resynthesis rate and instead generate heat [11,12]. NEFA also represent the primary substrate fueling BAT thermogenesis [13,14]. While intracellular BAT lipolysis has traditionally been recognized as the initial source of cold-stimulated NEFA production [11], recent evidence has shown that both dietary and white adipose tissue (WAT)-derived NEFAs can contribute significantly to thermogenesis [15]. Indeed, given the low thermogenic capability of human BAT—particularly in non-cold acclimated individuals—it is possible that dietary and WAT-derived NEFA are major substrates initiating and fueling cold-induced BAT thermogenesis.

NEFAs are a heterogenous class of lipids differing in chain length, degree of saturation and location of double bonds. There is ongoing speculation whether distinct NEFA species are integral in regulation of BAT activation and thermogenesis [7,11,16]. While the species-specific effects of NEFAs on BAT have not been described in human clinical studies, induction of *Ucp1* expression has been observed in cultured human pre-adipocytes stimulated with the omega-3 polyunsaturated NEFA eicosapentaenoic acid (EPA; C20:5 n3), while docosahexaenoic acid (DHA; C22:6 n3) and arachidonic acid (AA; C20:4 n6) were without effect [17]. Numerous rodent studies have shown that dietary omega-3 FA supplementation increases adipose tissue lipolysis, thermogenesis, *Ucp1* expression, UCP-1 protein concentration and BAT NEFA oxidation [18,19,20,21,22]. More recent studies have revealed the BAT-secreted linoleic acid-derived oxylipin (a family of oxidized lipids derived from NEFA), 12,13-dihydroxyoctadecenoic acid (12,13-diHOME), as a novel regulator of BAT activity [23,24]. Due to their low abundance and high volatility, the quantification of oxylipins has been limited, and it remains unclear whether the effects of 12,13-diHOME are species specific or shared by other oxylipins, NEFAs or divergent lipid species containing related side chains [7].

Given the preclinical evidence of NEFA and oxylipin involvement in BAT biology, we hypothesize circulating lipid species will be biomarkers for BAT activity or volume. In a cohort of healthy male subjects, we assess the association of plasma NEFA and oxylipins with cold-stimulated BAT activity and sympathetic nervous system activity. In addition, cold-stimulated changes in lipid concentrations were assessed for association with BAT activity.

## 2. Materials and Methods

This was a sub-study of a randomized, placebo-controlled, double-blinded clinical trial that has been previously described [9]. The current ancillary analysis explored the effect of acute cold exposure on BAT activity and the plasma lipidome in healthy individuals and was approved by the Alfred Health Ethics Committee and performed in accordance with the Declaration of Helsinki, Seventh Revision, 2013. All participants provided written, informed consent.

### 2.1. Participants

Fourteen healthy male participants (aged 19–30 years; BMI ≤ 25 kg/m^2^, free from cardiovascular disease and diabetes; unmedicated; sedentary; non-smokers) were recruited between June 2015 and July 2016. All participants first underwent screening that included a full medical history, physical examination, electrocardiogram (ECG), collection of fasting blood samples for baseline biochemical analysis and dual-energy X-ray absorptiometry (DEXA; GE Lunar iDXA; Getz Healthcare, West Melbourne, Vic, Australia) (Table 1 and as reported previously [9]).

### 2.2. Experimental Protocol

The experimental protocol was performed as previously described in detail [9]. Prior to the experimental visit, participants consumed a standardized meal between 18:00 and 22:00 h and were instructed to fast overnight, thereafter. Upon arrival at the laboratory at approximately 0800 h, participants rested quietly in a dimly lit room for 1.5 h with minimal mental and physical stimulation. A baseline (pre-cold) blood sample was collected, before participants were subjected to a standardized 90 min cold exposure via a temperature-controlled, water-perfused vest and blanket. The water perfusate temperature was adjusted to remain approximately 1 °C above the temperature that elicited mild shivering. Additional blood samples were collected at 30 min intervals during the cooling procedure.

Sixty min after the initiation of cooling, participants were injected with ^18^F-fluorodeoxyglucose ([^18^F]FDG), which was used to assess cold-stimulated BAT activity via positron emission tomography/computed tomography (PET/CT) imaging. Tracer uptake time was kept constant at 60 min, therefore imaging was initiated 60 min after injection and 120 min after cooling began, as previously described [9].

### 2.3. PET/CT Imaging

PET/CT imaging was conducted as previously described [9] and in accordance with recent guidelines [25]. Imaging was focused on the cervical and upper thoracic regions, bound superiorly by the base of the fourth cervical vertebra and inferiorly by the top of the third thoracic vertebra. Adipose tissues were determined based on a CT radiodensity between–190 and–10 Hounsfield units [25], and BAT was defined as a PET standardized uptake value (SUV), after correction for lean body mass, for any voxels within this defined region with a radiodensity of >1.5 [9,25]. BAT activity is most commonly reported as a composite calculation of intensity (mean SUV of all voxels meeting the required radiodensity and SUV threshold) multiplied by the total volume (BAT metabolic volume) of these voxels. Because both intensity of activity (SUVmean) and volume of activity (BAT metabolic volume) are relevant contributors to overall BAT activity, we examined both variables separately within this study to determine whether either factor independently associated with target lipids.

### 2.4. Biochemical Analysis

Blood samples were collected in appropriate tubes and measurements of fasting glucose, insulin, HbA1c (glycated hemoglobin), triglyceride, total, high- and low-density lipoprotein-associated cholesterol and thyroid hormones were performed immediately by the Alfred Hospital, Department of Pathology (Melbourne, Victoria, Australia). Further blood samples were collected in EDTA preservative tubes (BD Australia; North Ryde, NSW, Australia), centrifuged (1500× *g* for 15 min at 4 °C) within 10 min of collection, the plasma removed and frozen immediately at −80 °C for lipidomic analyses (described subsequently) and measurement of plasma norepinephrine (NE). NE was extracted using alumina adsorption, separated by high-performance liquid chromatography with colorimetric detection as previously described [26].

### 2.5. Lipidomic Analysis

Lipidomic analyses were conducted on plasma samples between 1 and 2 years after sample collection. While stability of the analytes was not evaluated in this study, previous publications have determined that long-term storage at −80 °C has minimal effect on oxylipin and NEFA concentrations [27,28,29], even in the absence of antioxidants. Reagents were purchased from Sigma-Aldrich (Sigma-Aldrich, Castle-Hill, NSW, Australia) unless specified. Plasma samples were prepared in a randomized order and interspersed with blanks (H_2_O) and pooled plasma quality controls (QCs). Lipids were extracted as previously described [30]. Briefly, 100 μL aliquots of plasma was transferred to 1.5 mL tubes containing 200 μL of 10% glycerol in water. Five μL of 10 mg/mL butylated hydroxytoluene (BHT) in ethanol was added to each tube, followed by 10 μL of an internal standard mix (Supplementary Table S1). Sample volumes were filled to 1.5 mL with 25% ACN in H_2_O and mixed thoroughly. Prepared samples were loaded into Oasis MAX SPE cartridges (Waters, Rydalmere NSW, Australia) according to the manufacturer’s instructions. Tubes were further rinsed with 1.5 mL of 25% ACN in H_2_O, which was added to the SPE cartridges. Cartridges were washed with 3 mL of 25% ACN in H_2_O, followed by 3 mL of ACN. Analytes were eluted with 1.3 mL of 1% formic acid in ACN into glass collection tubes containing 200 μL of 10% glycerol in methanol. Eluates were evaporated under a stream of nitrogen gas at 40 °C followed by reconstitution with 100 μL of MeOH/ACN (1:1). Reconstituted samples were transferred to a 0.2 mL glass insert inside 32 × 11.6 mm glass vials with Teflon insert caps for analysis.

Analysis of plasma extracts was performed on an Agilent 6495 QqQ mass spectrometer with an Agilent 1290 series high performance liquid chromatography (HPLC) system and a ZORBAX eclipse plus C18 column (2.1 × 100 mm, 1.8 μm, Agilent) with the thermostat set at 45 °C (Agilent Technologies Australia Pty, Ltd., Mulgrave, Vic, Australia). Mass spectrometry analysis was performed in negative ion mode with scheduled multiple reaction monitoring (MRM) or selected ion monitoring (SIM), where appropriate. Mass spectrometry settings and multiple reaction monitoring transitions are shown in Supplementary Table S2. The solvent system consisted of solvent A) 100% H_2_O containing 0.1% acetic acid and solvent B) 80% ACN/20% IPA (*v/v*) containing 0.1% acetic acid. Oxylipins and NEFA were eluted using a linear gradient from 20% solvent B to 100% solvent B over 15 min, followed by a 3 min wash at 100% solvent B and a 4 min re-equilibration at 20% solvent B. mass spectrometry source conditions were optimized using an internal standard mix. The following mass spectrometer conditions were used: drying gas temperature 230 °C, drying gas flow 15 L/min, nebulizer pressure 30 psi, sheath gas temperature 400 °C, sheath gas flow 10 L/min, capillary voltage 4000 V, nozzle voltage 1000 V, iFunnel high pressure RF voltage 130 V and iFunnel low pressure RF 80 V.

Lipid concentrations were calculated by relating the peak area of each species to the peak area of the corresponding internal standard. Peak integration was performed using MassHunter quantitative analysis B.09.00 (Agilent Technologies). If any given transition yielded multiple distinguishable peaks (due to the presence of isomers and branched variants), each peak was treated as a separate species and analyzed accordingly. Analytical reproducibility was determined by calculating the coefficient of variation for each lipid using pooled plasma quality controls. Lipid concentrations are reported as pmol/mL of plasma.

### 2.6. Statistical Analyses

Total NEFA concentration was calculated by summing the concentrations of each NEFA species. Peak experimental NE concentrations were defined as the highest NE concentration recorded while cold-exposed (at either 30, 60 or 90 min after initiation of cooling). Peak NE concentration change compared to basal NE concentration was employed as a surrogate index of sympathetic activation induced by the bout of cold exposure. All parameters were assessed for normality using the Shapiro–Wilk test. Data were determined to be non-normal for many lipid species and therefore nonparametric analyses were applied. Given the high degree of collinearity between plasma concentration of individual NEFA and oxylipin species and our focus on discovery, false discovery rate correction was not applied to limit false negatives.

Pre- and post-cold total lipid concentrations (both individual species and total NEFA concentration) were compared with a Wilcoxon signed-rank test. The total NEFA concentration change (% change from baseline) was correlated against post-cold exposure BAT activity (SUVmean), BAT metabolic volume and peak NE concentration change using Pearson’s correlation coefficient. Statistical analyses were conducted using R (v3.5.3) and *p* < 0.05 was deemed statistically significant.

## 3. Results

The study cohort baseline characteristics are reported as mean and standard deviation in Table 1, and the cohort is identical to that previously reported by us [9].

### 3.1. Cold Exposure Increases Total NEFA Concentration in Association with Increased Plasma NE

**Figure 1:**
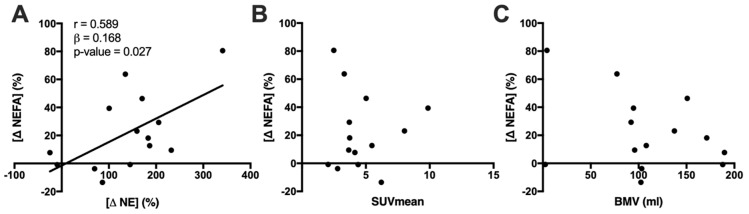
Correlation of the change in total plasma non-esterified fatty acid (NEFA) concentration after 90 min mild cold exposure against (**A**) peak norepinephrine (NE) concentration change and (**B)** brown adipose tissue (BAT) activity (SUVmean; (**C**) BAT metabolic volume (BMV). Pearson’s r value (*n* = 14) is shown for significant correlations (*p* < 0.05).

Collectively, 44 individual NEFA species and 41 individual oxylipin species were quantified in this lipidomic analysis (each species and their absolute concentrations are shown in Supplementary Table S3). Cold exposure increased total NEFA concentration by 22.2% (*p* = 0.007). Change in total NEFA concentration (range: −13.49–80.52%) was significantly correlated with the peak NE concentration change (range: −24.98–340.95%) (Figure 1A), but not with indices of BAT activity (Figure 1B,C). Total oxylipin concentration did not significantly change following cold exposure (change = 4.97%, *p* = 0.241, 95% CI −2.88, 12.82), nor did it correlate with changes in either BAT activity (*r* = 0.338, *p* = 0.237) or NE concentration (*r* = 0.526, *p* = 0.053).

### 3.2. Cold Exposure Increases Individual NEFA Species Concentration

**Figure 2:**
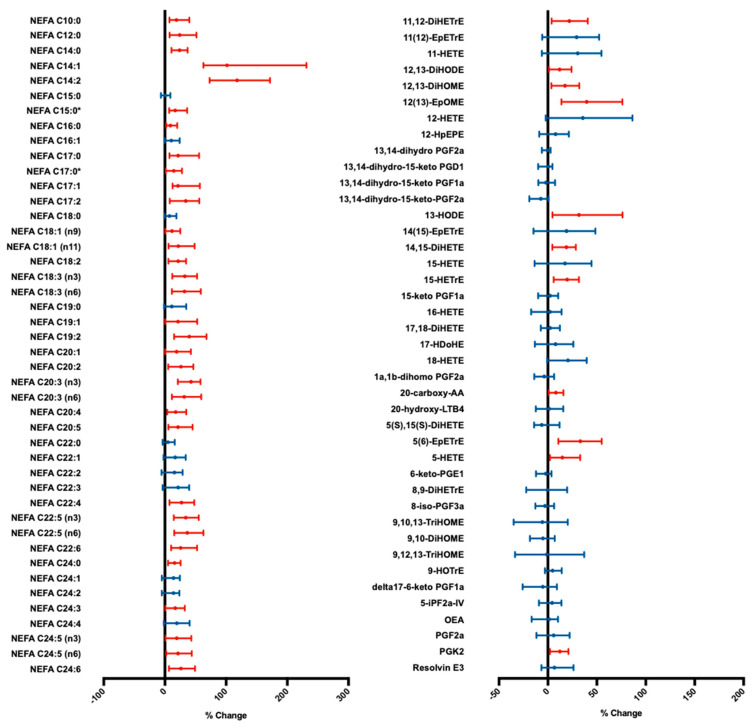
Forest plot of median non-esterified fatty acid (NEFA; left panel) and oxylipin (right panel) species plasma concentration change during cold exposure. Error bars indicate 95% confidence intervals. *p* values calculated using a Wilcoxon signed-rank test (*n* = 14). Significant changes (*p* < 0.05) shown in red; statistically insignificant changes shown in blue. * Indicates branched isomers. DiHETrE—dihydroxyeicosatrienoic acid; EpETrE—epoxyeicosatrienoic acid; DiHODE—dihydroxyoctadecadienoic acid; DiHOME—dihydroxyoctadecenoic acid; EpOME—epoxyoctadecenoic acid; HETE—hydroxyeicosatetraenoic acid; HpEPE—hydroperoxyl-eicosapentaenoic acid; PG—prostaglandin; HODE—hydroxyoctadecadienoic acid; DiHETE—dihydroxy-eicosatetraenoic acid; HDoHE—hydroxy-docosahexaenoic acid; AA—arachidonic acid; LT—leukotriene; HOTrE—hydroxyoctadecatrienoic acid; TriHOME—trihydroxyoctadecenoic acid; iP—isoprostane; OEA—oleoylethanolamine.

Individually, 33 NEFA species changed significantly in concentration with cold exposure, all of which increased relative to baseline (Figure 2). In particular, the largest increases relative to baseline were observed with NEFA C14:1 and C14:2, increasing by 102% (*p* < 0.001, 95% CI 63, 231) and 118% (*p* < 0.001, 95% CI 73, 171), respectively. Similarly, 11 oxylipin species significantly increased in concentration with cold exposure (Figure 2).

### 3.3. NEFA Species Concentrations Are Correlated with Cold-Stimulated BAT Activity

**Figure 3:**
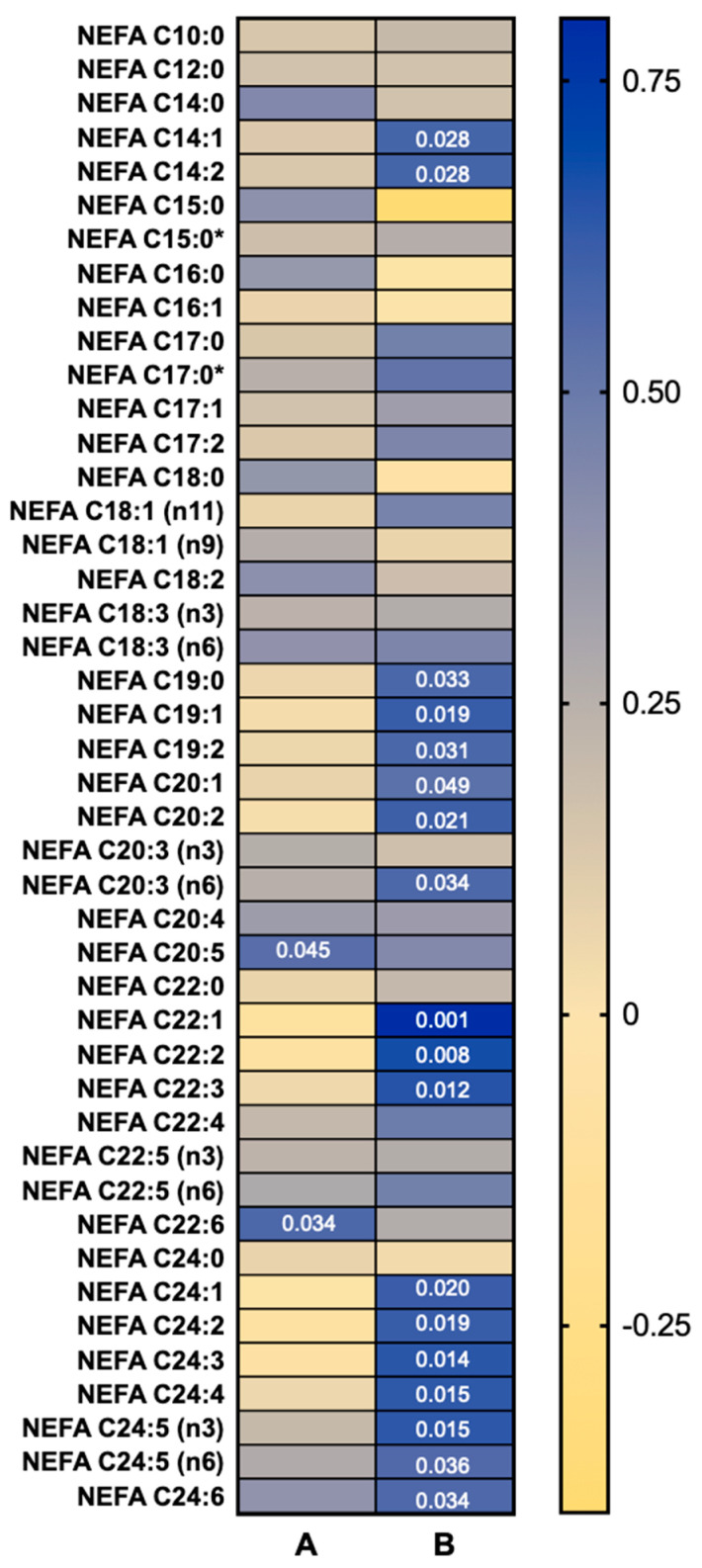
Heat map of Pearson’s *r* values of correlations between (**A**) plasma non-esterified fatty acid (NEFA) species baseline concentration and (**B**) concentration change after 90 min mild cold exposure against brown adipose tissue (BAT) activity (SUVmean) (*n* = 14). Significant correlations (*p* < 0.05) indicated by the *p* values within the heat map. * Indicates branched isomers.

Correlations between concentrations of individual NEFA species at baseline and cold-induced changes in BAT SUVmean are shown in Figure 3; correlations against BAT metabolic volume are outlined in Supplementary Table S3. The baseline (pre-cold) concentrations of two NEFA species, NEFA C20:5n3 (EPA) and C22:6n3 (DHA), were positively correlated with BAT SUVmean (Figure 3A), but not BAT metabolic volume (Supplementary Table S3). The cold-induced concentration changes of 18 NEFA species were positively correlated with BAT SUVmean (Figure 3B).

Neither the baseline concentrations nor changes with cold exposure of individual oxylipin species correlated with BAT SUVmean. However, concentration changes in six oxylipin species were correlated with BAT metabolic volume (Supplementary Table S3).

## 4. Discussion

It is well established that NEFAs mobilized by sympathetic stimulation are both primary substrates for oxidation—as well as activators of UCP-1 for BAT thermogenesis [7]. However, there is limited evidence regarding whether there are species specific relationships between individual NEFAs and BAT activity in humans. The primary observations in the present study are the associations between cold-stimulated BAT activity and the resting concentrations of two clinically important n-3 polyunsaturated NEFA species. This represents the first association in humans to support existing preclinical data for involvement of NEFA in regulation of cold-stimulated BAT activity, warranting further investigation of n-3 polyunsaturated NEFA species as potential therapeutic BAT activators. Furthermore, cold-stimulated BAT activity positively correlated with concentration change of 18 NEFA species, but not total NEFA concentration change, suggesting potential species-specific effects of BAT activity on the circulating NEFA profile.

The present study observed that fasting, basal concentrations of n-3 polyunsaturated NEFA species, EPA and DHA, were positively correlated with cold-stimulated BAT activity. EPA and DHA have been of scientific interest for several years due their various associated health benefits [31]. Notably, dietary EPA and DHA supplementation has been shown to increase adipose tissue lipolysis, thermogenesis, *Ucp1* expression, UCP-1 protein concentration and BAT NEFA oxidation in rodents [18,19,20,21,22]. Similarly, EPA treatment has been shown to induce *Ucp1* expression in cultured human pre-adipocytes [17]. Although the capacity for EPA and DHA treatment to meaningfully increase BAT activity has not been assessed in human clinical trials, high dietary n-3 polyunsaturated NEFA intake has been associated with the attenuation of genetically driven weight gain in large cohort studies [32], with increased BAT activity a plausible etiological contributor. Furthermore, treatment with a purified EPA ethyl ester (icosapent ethyl) was recently shown to reduce the risk of major cardiovascular events by 28% among hypertriglyceridemic individuals [33]. Nevertheless, the present findings cannot establish whether the associations between BAT activity and circulating EPA and DHA concentrations are mechanistic or associative. Preclinical studies support a direct mechanistic role of EPA and DHA in the upregulation BAT activity in humans [17,18,19,20,21,22], however indirect relationships as a result of NEFA oxidation may also have value as potential biomarkers of BAT activity. Further human studies including interventions and NEFA tracers are required to fully elucidate the relationship between n-3 NEFAs and BAT activity.

A consensus model of cold exposure proposes that it leads to sympathetically mediated NEFA mobilization [34], predominantly via white adipose tissue lipolysis [35,36]. A portion of mobilized NEFA are taken up by BAT, leading to activation of UCP-1 and provision of substrates for thermogenesis [15,37,38]. Here, we observed a positive correlation between peak NE concentration change (a surrogate marker of sympathetic activation in response to cold) and total NEFA concentration change. Cold-induced increases in 18 NEFA species were significantly positively correlated with BAT activity, despite no significant correlation between total NEFA concentration change and BAT activity. Taken together, these observations are consistent with a generalized lipolytic response to a sympathetic stimulation, but also suggest a species-specific association of NEFA with BAT activation. NEFA species unaffected by cold exposure may serve to fuel other thermogenic tissues or be used in hepatic TG [34,35,39] and acylcarnitine [40] synthesis.

The distinct NEFA species that positively correlate with BAT activity could reflect those preferentially taken up and metabolized by BAT, as has been observed with other tissues [41,42]. The robust elevation in plasma C14:1 and C14:2 NEFA following cold exposure could suggest an upregulation of fatty acid beta–oxidation. These species are produced following two cycles of beta–oxidation of the abundant C18:1 and C18:2 NEFA species. This is consistent with an increase in BAT NEFA oxidation as a response to cold-induced thermogenesis [43,44]. The observation of significant cold-induced concentration changes in most odd-chain NEFA is also of interest given the evolving understanding of odd-chain lipid synthesis and metabolism in humans [45,46]. Previously thought to be strictly prokaryote-derived, it is now recognized that odd-chain fatty acids can be obtained through diet [47], produced by intestinal microbiota activity [48], endogenously synthesized from propionic acid, alpha–oxidation of hydroxylated fatty acids or produced from branched chain amino acids [49,50]. Interestingly, BAT was shown to exhibit high rates of branched-chain fatty acid synthesis [50]. Given that identification of endogenous metabolism of these species is relatively recent, the factors that influence their production, transport and storage, is not well understood.

In contrast to a BAT-specific perspective of the observed relationships, it is important to consider alternative explanations. A majority of these NEFA were very-long chain acyl species with 1–3 double bonds. These cover a broad spectrum of endogenously synthesized species—as well as those produced from essential NEFA. We also cannot rule out the possibility that an individual’s BAT activity is confounded by long-term dietary intake. Indeed, circulating fatty acid composition has been shown to correlate with dietary intake [51]. Furthermore, adipose fatty acid composition has been used as a surrogate for long-term dietary assessment [52]. Therefore, cold-stimulated NEFA release may reflect long-term dietary lipid composition and white adipose lipolysis. It is noteworthy that these hypotheses presume a relative non-selectivity in BAT NEFA substrates that would confer an evolutionary survival advantage, albeit with the likelihood that a hierarchy for preference exists within this relative non-selectivity [7]. However, the present study cannot, distinguish between either the source or final destination for any NEFA species. Overall, these observations therefore highlight the need for dedicated intervention studies and serve to highlight the dearth of understanding of the role of dietary fatty acids in regulation of human BAT activity.

Associations observed between lipid concentrations and BAT activity, in the present study, were primarily restricted to the intensity of BAT activity (SUVmean), but not BAT metabolic volume. Although this could partially be due to the large range in BAT metabolic volume in the present cohort, recent evidence indicates a broad range for BAT metabolic volume is reflective of the norm for healthy mild-cold exposed humans [1,53]. Therefore, for the purpose of the present analysis, we focused only on level of activity within a targeted region (SUVmean) without highly variable tissue volume as a confounding factor. In the present model, a given local concentration of NEFA will activate functional BAT depots, regardless of their size and their interaction with surrounding “whiter” depots. Consequently, this would confound the analysis through accounting for a highly variable factor (BAT volume) while disproportionately reducing the weighting of the most relevant factor (intensity of activity in regions of metabolically active BAT). From a therapeutic perspective, the present analysis may only be useful to detect lipid-related molecules that drive thermogenesis in highly cold-adapted BAT.

Recently, 12,13-diHOME was identified as a BAT-secreted oxylipin with autocrine, paracrine [23] and endocrine [24] effects. Fasting plasma 12,13-diHOME concentrations have been inversely correlated with adiposity and fasting insulin and triglyceride levels [54]. However, while a 20.3% increase in 12,13-diHOME was observed following cold-exposure (Figure 2), this was not associated with BAT activity (Supplementary Table S3). Furthermore, this study did not observe any significant associations between either the baseline concentrations or cold-induced concentration changes of any oxylipin species examined (Supplementary Table S3). This may be explained by the low abundance and high volatility of oxylipin species, compounded with a small sample size and minor, but potentially relevant differences in protocol (duration, method and temperature of cooling), impeding the observation of small BAT-derived oxylipin alterations. There is also evidence to suggest that the flux of 12,13-diHOME and other oxylipins is largely driven by fluctuating dietary availability [55] and the relative abundance of respective “parent” NEFA [56] rather than the selective activity of a particular thermogenic tissue. Nevertheless, BAT oxylipin metabolism requires further study—particularly in humans.

### Limitations

As is the case for previous human BAT clinical studies, the present investigation was limited by the use of [^18^F]FDG PET/CT imaging to determine the extent of BAT activity. As thoroughly described by several groups (for a recent comprehensive review see [57]), including us [9,16], [^18^F]FDG PET/CT imaging measures BAT glucose uptake as a surrogate for BAT energy expenditure. However, it is known these two variables can become disconnected under various physiological [58,59], pathologic [13] and interventional conditions [60], such that changes in BAT [^18^F]FDG uptake ceases to represent a surrogate of BAT thermogenesis. This method underestimates total BAT activity due to not including the contribution of lipid substrates [7] and it cannot account for potential substrate switching within adipocytes, induced by certain interventions, which may be independent of total energy expenditure [9]. Related to this is the potential for, but as yet unknown in humans, relevance of beige adipocytes with a preference for one substrate over another [61]. These factors are particularly significant given the present focus on NEFA. Nevertheless, the search for lipid factors that influence BAT thermogenic activity involving glucose metabolism remains relevant. Future studies should search for lipid (or otherwise) biomarkers and BAT activators that influence the separate components of BAT energy metabolism.

Given the relatively minor contribution that human BAT makes to whole body lipid metabolism, it is possible that other tissues, such as white and “beige’ adipose tissue, skeletal muscle, cardiac tissue and liver [13,62], may “mask” BAT-specific metabolic signatures. This may have been further exacerbated by the use of venous plasma. Moreover, individual differences in physical fitness, muscle mass, sympathetic and adrenergic signaling, adiposity and adipose tissue subtype mix—as well as diet—can all effect observed patterns in lipid flux and warrants further clinical investigation. While BAT has the highest cold-stimulated glucose uptake per volume of tissue [14,63], the average young healthy male is expected to have >50 times greater volume of skeletal muscle than BAT [1,64]. Similarly, estimates of BAT NEFA uptake based on radiolabeled NEFA studies suggest that BAT contributes to <1% of whole-body NEFA uptake [14,65]. Future clinical studies should first seek to compare BAT-derived lipid modulation before and after prolonged periods of cold- adaptation, in order to better elucidate BAT-specific effects. Tracer methodologies—as well as novel techniques such as microdialysis—that allow for the comparison of localized tissue substrate changes in vivo, represent additional strategies to examine tissue-specific sympathetic activity and lipid flux in future studies [66]. Future studies may also consider an extended period of diet standardization prior to experimentation, including the potential division of participants into dietary subsets (for example, variation in fat and carbohydrate content), to better delineate diet-dependent and diet-independent observations. Examination of the gastrointestinal microbiome and measurement of circulating lipopolysaccharide (LPS), may allow greater understanding of the role of gut permeability and inflammation in lipid metabolism, its modulation by obesity and metabolic syndrome and any potential link to BAT physiology.

## 5. Conclusions

The importance of the search for factors that are endogenous biomarkers and potent regulators of BAT browning and thermogenesis remains. Moreover, recent trends that highlight species (human versus mouse) divergence in BAT physiology illustrate the urgent need to focus attention on humans. This study presents associative data from humans, supporting preclinical studies that have previously highlighted that further attention is warranted for therapeutic development of n-3 NEFAs in recruitment of BAT. Additional relationships observed suggest that beyond the well-studied n-3 species of EPA and DHA, other NEFAs or indeed other structurally distinct lipids, may be relevant. Future studies should be designed to detect BAT-specific interactions with target lipids. Moreover, the current indeterminate relationship between lipids and BAT metabolism argues for the net to be cast more widely in the search for clinical activators and biomarkers of BAT.

## Figures and Tables

**Table 1:** Participant baseline characteristics (*n* = 14).

Participant Characteristics	Mean	SD
**Physical Characteristics**		
Age (years)	21.8	2.1
Height (cm)	176.1	7.6
Weight (kg)	66.2	9.0
BMI (kg/m^2^)	21.3	2.2
Body fat (%)	19.5	4.6
**Fasting Plasma Hormones, Lipids and Metabolites**		
Glucose (mmol/L)	4.9	0.2
Insulin (mU/L)	6.0	2.9
HbA1c (%)	5.3	0.2
Total cholesterol (mmol/L)	4.0	0.5
HDL-C (mmol/L)	1.3	0.2
LDL-C (mmol/L)	2.4	0.5
TG (mmol/L)	0.7	0.1
NE (pg/mL)	234	88
TSH (mU/L)	1.3	0.7
T3 (pmol/L)	4.7	0.5
T4 (pmol/L)	13.4	1.4

SD—standard deviation; BMI—body mass index; HbA1c—glycated hemoglobin; HDL-C—high-density lipoprotein cholesterol; LDL-C—low-density lipoprotein cholesterol; TG—triglycerides; NE—norepinephrine; TSH—thyroid stimulating hormone; T3—triiodothyronine; T4—thyroxine.

## Data Availability

Targeted lipidomics profiling was performed at the Metabolomics Laboratory (Dr. P. Meikle), Baker Heart and Diabetes Institute, Melbourne, Victoria, Australia, where the raw data are held.

## Supplementary Materials

The following are available online at https://www.mdpi.com/2218-1989/10/10/388/s1, Table S1: List of internal standards used for quantitation, Table S2: MRM transitions and retention times, Table S3: List of lipid species and regression analyses against BAT activity and volume.

## Abbreviations

BAT—brown adipose tissue; NEFA—non-esterified fatty acid; [18F]FDG—18F-fluorodeoxyglucose; PET—positron emission tomography; CT—computed tomography; NE—norepinephrine; DHA—docosahexaenoic acid; EPA—eicosapentaenoic acid; UCP-1—uncoupling protein-1; WAT—white adipose tissue; 12,13-diHOME—12,13-dihydroxyoctadecenoic acid.
